# A Systematic Review to Clarify the Prognostic Values of CD44 and CD44^+^CD24^-^ Phenotype in Triple-Negative Breast Cancer Patients: Lessons Learned and The Road Ahead

**DOI:** 10.3389/fonc.2021.689839

**Published:** 2021-08-09

**Authors:** Mahdi Abdoli Shadbad, Negar Hosseinkhani, Zahra Asadzadeh, Afshin Derakhshani, Noora Karim Ahangar, Nima Hemmat, Parisa Lotfinejad, Oronzo Brunetti, Nicola Silvestris, Behzad Baradaran

**Affiliations:** ^1^Research Center for Evidence-Based Medicine, Faculty of Medicine, Tabriz University of Medical Sciences, Tabriz, Iran; ^2^Immunology Research Center, Tabriz University of Medical Sciences, Tabriz, Iran; ^3^Student Research Committee, Tabriz University of Medical Sciences, Tabriz, Iran; ^4^Laboratory of Experimental Pharmacology, IRCCS Istituto Tumori Giovanni Paolo II, Bari, Italy; ^5^Medical Oncology Unit, Istituto di Ricovero e Cura a Carattere Scientifico (IRCCS) Istituto Tumori Giovanni Paolo II, Bari, Italy; ^6^Department of Biomedical Sciences and Human Oncology, Aldo Moro University of Bari, Bari, Italy; ^7^Department of Immunology, Faculty of Medicine, Tabriz University of Medical Sciences, Tabriz, Iran

**Keywords:** triple-negative breast cancer, cancer stem cell, cancer therapeutic resistance, CD44, CD44/CD24, prognosis

## Abstract

As a unique population of tumor bulk, cancer stem cells have been implicated in tumor relapse and chemoresistance in triple-negative breast cancer (TNBC). Therefore, understanding the phenotype of cancer stem cells can pave the way for introducing novel molecular targeted therapies for treating TNBC patients. Preclinical studies have identified CD44^+^CD24^-/low^ as a cancer stem cell phenotype; however, clinical studies have reported seemingly controversial results regarding the prognostic values of CD44 and CD44^+^CD24^-/low^ phenotype in TNBC patients. To critically review the clinicopathological significance and prognostic values of CD44 and CD44^+^CD24^-/low^ phenotype in TNBC patients, the Scopus, Embase, PubMed, and Web of Science databases were systematically searched to obtain the relevant records published before 20 October 2020. Based on nine included studies, CD44 and CD44^+^CD24^-/low^ phenotype are associated with inferior prognosis in TNBC patients. Moreover, these cancer stem cell markers have been associated with advanced tumor stage, tumor size, higher tumor grade, tumor metastasis, and lymphatic involvement in TNBC patients. Our evidence has also indicated that, unlike the treatment-naïve TNBC patients, the tumoral cells of chemoradiotherapy-treated TNBC patients can upregulate the CD44^+^CD24^-/low^ phenotype and establish an inverse association with androgen receptor (AR), leading to the inferior prognosis of affected patients. In summary, CD44 and CD44^+^CD24^-/low^ phenotype can be utilized to determine TNBC patients’ prognosis in the pathology department as a routine practice, and targeting these phenotypes can substantially improve the prognosis of TNBC patients.

## Introduction

Breast cancer is one of the frequently diagnosed cancers among females (1). TNBC, as one of the troublesome breast cancer subtypes, is characterized by the lack of expression of estrogen receptor, progesterone receptor, and human epidermal growth factor receptor 2 (HER2) (2). TNBC can be further grouped into six subtypes, i.e., basal-like 1, basal like2, immunomodulatory, mesenchymal, mesenchymal stem-like, and luminal androgen receptor subtypes (3). Despite recent advances in treating breast cancer, the current therapeutic approaches have not resulted in desirable outcomes for TNBC patients. Therefore, there is a need to develop new approaches to treat TNBC patients (3).

Although cancer stem cells comprise a small tumor cell population, their self-renewal feature can facilitate rising progressive neoplasms. This unique tumor cell population is one of the culprits of developing chemoresistance and tumor relapse (4). Indeed, cancer stem cells share many features with normal stem cells; for instance, they can be divided asymmetrically and recapitulate tumor cells (5). Furthermore, cancer stem cells can stimulate the epithelial-to-mesenchymal transition (EMT) process to facilitate tumor metastasis (6).

Preclinical studies have indicated that CD44, as a transmembrane glycoprotein, is overexpressed in cancer stem cells and has been implicated in tumor development and migration (7, 8). The interaction between CD44 and hyaluronan can stimulate the epidermal growth factor receptor (EGFR)-related pathways and facilitate chemoresistance, tumor growth, and metastasis in various cancers (9). Indeed, CD44 has been implicated in the activation of the mitogen-activated protein kinase (MAPK)/extracellular signal-regulated kinase (ERK) and the phosphatidylinositol-3-kinase and protein kinase B (PI3K/Akt) signaling pathways in tumoral cells (10, 11). The activation of the rat sarcoma (Ras)- rapidly accelerated fibrosarcoma (Raf)-extracellular signal-regulated kinase kinase (MEK)-ERK pathway has been associated with upregulated tumoral programmed death-ligand 1 (PD-L1) expression, which ultimately establishes an auto-inductive loop with PD-L1 (12–14). Therefore, CD44 can facilitate the immune evasion of tumoral cells *via* facilitating the activation of the Ras-Raf-MEK-ERK pathway. Indeed, recent findings have indicated that CD44 can promote the expression of tumoral PD-L1 in TNBC cells (15). Nam et al. have indicated that CD44 can promote the activation of the tyrosine-protein kinase Src (c-Src)/Akt signaling pathway, leading to the activation of c-Jun and transcription of c-Src. Therefore, CD44-mediated c-Src/Akt/c-Jun/c-Src signaling pathway can lead to the establishment of an auto-inductive, resulting in tumorigenesis and migration in breast cancer cells (16). Furthermore, the interaction of CD44 with its ligand, hyaluronic acid, has upregulated expression of multidrug resistance 1 (MDR-1) in Nanog/signal transducer and activator of transcription (STAT)-3-mediated fashion (17). The upregulation of STAT-3 has also been associated with increased expression of matrix metalloproteinase-2 (MMP)-2 and invasion in tumoral cells (18). Besides, CD44 can provide an activation site for Ezrin-Radixin-Moesin, leading to cytoskeletal modifications and migration (19). Therefore, preclinical studies have indicated CD44 has been implicated in tumorigenesis, chemoresistance, immune evasion, and migration in cancers.

In 2003, Al-Hajj et al. indicated that the CD44^+^/CD24^-^/Lin^-^ phenotype can be linked to cancer stem cell features in breast cancer (20). In line with this, Taniuchi et al. have indicated that CD24 can inhibit the migration and metastasis of pancreatic cancer cells (21). Moreover, it has been reported that CD24 is less expressed in differentiated cells compared to progenitor cells (22). Pallegar et al. have shown that the activation of Raf can substantially downregulate the gene and protein expression of CD24 (23). Moreover, the activation of Ras has been associated with the generation of CD44^+^/CD24^-^ cells from the CD44^-^/CD24^+^ cells in breast cancer (24). Consistent with these, recent data have shown that inhibiting ERK, which belongs to the Ras-Raf-MEK-ERK pathway, can substantially decrease the population of cells with CD44^+^/CD24^-^ in TNBC (10). Thus, preclinical studies indicate that the CD44^+^/CD24^-^ phenotype can be associated with tumor development and migration in breast cancer cells. However, the published clinical studies have not reached a consensus regarding the prognostic value of these phenotypes in TNBC patients (25–29).

Therefore, there is a need to clarify the prognostic role and clinical significance of these phenotypes in TNBC patients. This systematic review aimed to discuss the prognostic role and clinicopathological relevance of CD44 and CD44^+^CD24^-/low^ phenotype in TNBC patients. Furthermore, this study intended to briefly review novel approaches to target CD44 to ameliorate the prognosis of TNBC patients.

## Methods

This study was conducted under the preferred reporting items for systematic reviews and meta-analyses (PRISMA) statements (30).

### The Strategy of the Systematic Search

The Scopus, PubMed, Web of Science, and Embase databases were systematically searched to obtain the relevant studies published before 20 October 2020. For this purpose, the abovementioned databases were systematically searched with the following keywords: (“CD44” OR”CD 44” OR “HCAM” OR “homing cell adhesion molecule” OR “Pgp-1” OR “phagocytic glycoprotein-1” OR “phagocytic glycoprotein 1” OR “phagocytic glycoprotein1” OR “Hermes antigen” OR “lymphocyte homing receptor” OR “ECM-III” OR “HUTCH-1” OR “H-CAM” OR “Ly-24” OR “Cluster of Differentiation 44” OR “Cluster of Differentiation44”) and (“TNBC” OR “triple-negative” OR “triple negative” OR “triple-negative breast cancer” OR “triple negative breast cancer” OR “ER-negative PR-negative HER2-negative breast neoplasms” OR “ER negative PR negative HER2 negative breast neoplasms” OR “triple-negative breast cancers” OR “triple-negative breast neoplasm” OR “triple negative breast neoplasm” OR “triple-negative breast neoplasms” OR “ER-negative PR-negative HER2-negative breast cancer” OR “ER negative PR negative HER2 negative breast cancer” OR “triple negative breast cancer”).

### Study Selection and Data Extraction

After the systematic search, the obtained studies were reviewed in two phases. In phase I, two authors (N.H and Z.A) independently screened records according to their titles and abstracts. In phase II, the same authors independently reviewed the full text of the remaining papers, along with their supplementary data. Any disagreements were resolved *via* consulting with B.B and consensus.

### Data Extraction

The following data were extracted from the included studies: (1) the first author, (2) publication year, (3) the country, (4) the sample size, (5) the previous treatment of affected patients, (6) the prognostic values of CD44-CD44^+^/CD44^-/low^ phenotype, e.g., progression-free survival (PFS), overall survival (OS), disease-free survival (DFS), breast cancer-specific survival (if reported), (7) the association between CD44-CD44/CD44 phenotypes with the clinicopathological features, and (8) the association between CD44-CD44/CD44 phenotypes with the EMT/metastasis factors.

### Eligibility Criteria

Papers with the following eligibility criteria were included in our study: (1) human-based studies, (2) investigations with the objective of assessing the CD44-CD44^+^/CD44^-/low^ phenotype in TNBC patients, (3) studies, which investigated the protein expression of CD44 and CD44^+^/CD44^-/low^ phenotype TNBC patients, (4) studies, which demonstrated the prognostic value of CD44-CD44^+^/CD44^-/low^ phenotype or the association between the clinicopathological characteristics with CD44-CD44^+^/CD44^-/low^ phenotype in patients with TNBC, and (5) studies, which were published in English. Based on the following criteria, records were excluded from this study: (1) studies that failed to meet the aforementioned inclusion criteria, (2) duplicated studies, (3) review papers, (4) studies, which did not evaluate the protein expression of CD44-CD44^+^/CD44^-/low^ phenotype, rather the gene expression, (5) conference abstracts, (6) cellular studies, and (7) animal studies.

### Risk of Bias in Included Studies

The methodologies of included investigations were assessed using Hayden et al. guidelines for assessing the quality of our included studies (31). Any disagreements were resolved *via* consulting with B.B. The evaluation is demonstrated in **Table 1**.

**Table 1:** The risk of bias assessment based on the Hayden et al. statements.

First author, year	Study participation	Study attrition	Prognostic factor measurement	Outcome measurement	Confounding measurement and account	Analysis
**Diego de Mendonca Uchôa, (** 32 **)**	***	***	***	**	***	**
**Francesca Collina, (** 25 **)**	***	***	**	**	*	**
**Min Hye Jang, (** 33 **)**	***	***	***	**	***	**
**Shu-Jyuan Chang, (** 34 **)**	***	***	*	***	***	**
**Fang Yang, (** 26 **)**	***	***	*	***	***	**
**Yan−Xi Liu, (** 27 **)**	***	***	**	***	***	**
**Hui Wang, (** 28 **)**	***	***	**	***	***	**
**Weiyan Zou, (** 29 **)**	***	***	***	**	**	***
**Nazia Riaz, (** 35 **)**	***	***	***	***	***	***

***, no bias might exist; **, Partly bias might exist, and *, bias might exist.

## Results

### Selected Studies

**Figure 1:**
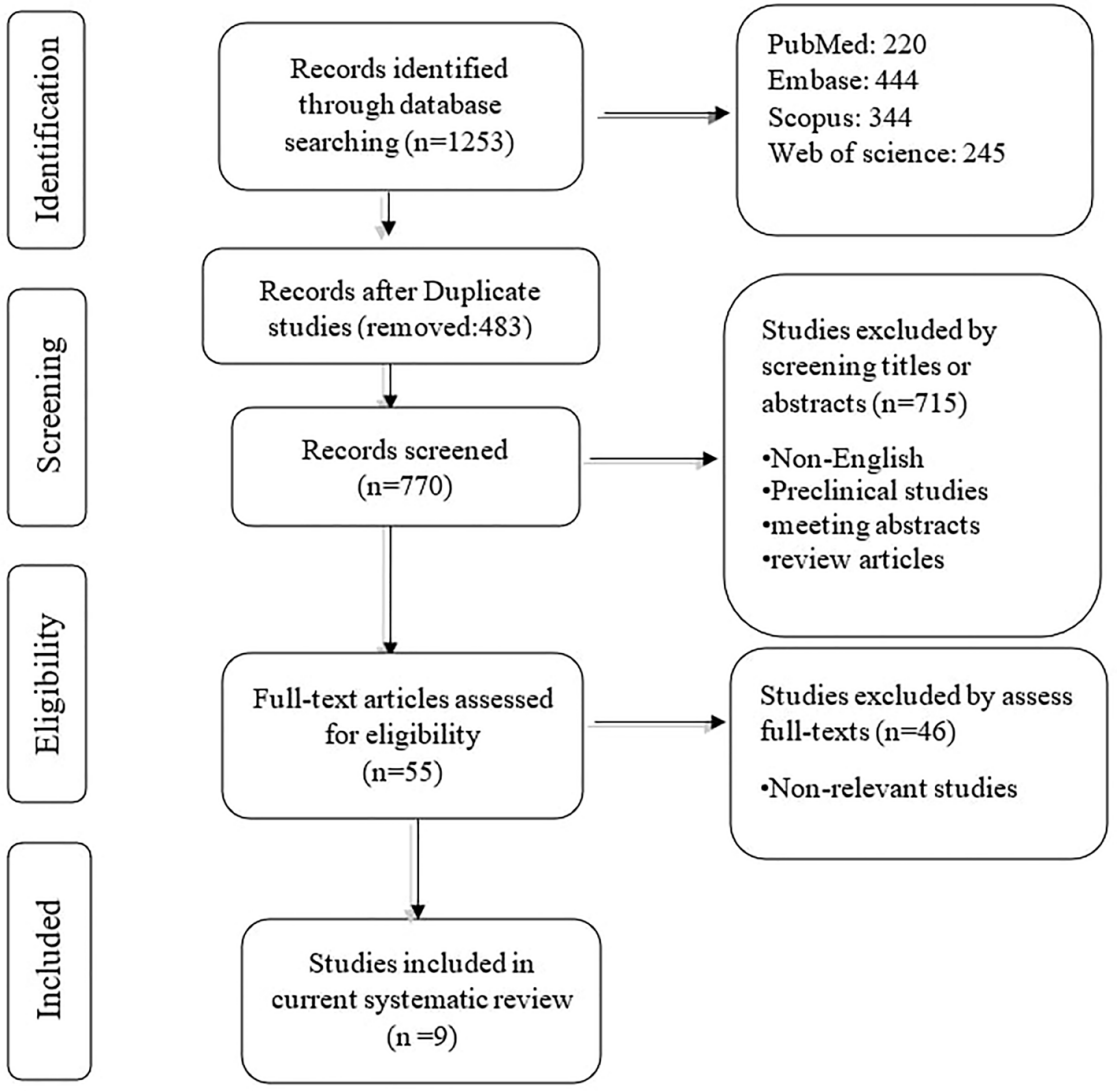
Flowchart of the study selection process.

The systematic search retrieved 1253 records: PubMed (220), Embase (444), Scopus (344), and Web of Science (245). After removing duplication records, 770 records remained. In phase I, 715 studies were removed based on reviewing the title/abstract of the remaining records. In phase II, two authors reviewed the full text of 55 remaining studies, along with their supplementary data. Based on the second phase of reviewing, nine papers were included in the qualitative synthesis. The flowchart of literature identification, inclusion, and exclusion is demonstrated in **Figure 1**.

### The Characteristic of Included Studies

The nine clinical studies were published in English between 2014 and 2020. All investigations utilized immunohistochemistry (IHC) as the staining method. Regarding the clinico- pathological significance of CD44 in TNBC patients, CD44 has been associated with lymphovascular invasion, metastasis, higher tumor grade, lymph node metastasis, and advanced tumor stage in patients with TNBC (**Table 2**). Regarding the clinicopathological significance of CD44^+^CD24^-/low^ phenotype in TNBC patients, CD44^+^CD24^-/low^ phenotype has been associated with tumor grade, tumor stage, tumor size, histology classification, lymph node metastasis, and AR expression; however, this phenotype has been inversely associated with AR expression in TNBC patients treated with chemotherapy/radiotherapy (**Table 2** for a better elucidation, refer to the discussion).

**Table 2:** The clinicopathological significance of CD44 and CD44^+^CD24^-/low^ phenotype in TNBC patients.

First author, year	Number of patients	Studied factor	Clinical significance	Cut-off for considering as positive/overexpressed	Evaluation method	The reference ID of used antibody for IHC	Number of patients with previous treatment
**Diego de Mendonça Uchôa, (** 32 **)**	47	CD44	It is negatively associated with lymphovascular invasion.	Above 1%	IHC	MRQ-13 for CD44	N/A
**Francesca Collina, (** 25 **)**	143	CD44	It is associated with metastasis.	Above 50%	IHC	No reference ID	N/A
**Weiyan Zou, (** 29 **)**	51	CD44	It is associated with higher tumor grade, large tumor size, increased lymph node metastasis, and advanced tumor stage.	N/A	IHC	ab51037 for CD44	N/A
**Diego de Mendonça Uchôa, (** 32 **)**	47	CD44^+^CD24^-/low^	It is associated with increased tumor size.	For CD44, it was 1%, and for CD24, it was 33%.	IHC	MRQ-13 for CD44, and SN3b for CD24	N/A
**Min Hye Jang, (** 33 **)**	172	CD44^+^CD24^-/low^	It is associated with high tumor grade.	For CD44, it was 10% and above, and for CD24, it was 10% and above.	IHC	156-3C11 for CD44, and SN3b for CD24	N/A
**Shu-Jyuan Chang, 2015**	67	CD44^+^CD24^-/low^	No statistically significant associations were found (P-values > 0.05)	Not appropriately provided	IHC	No reference ID	N/A
**Fang Yang, (** 26 **)**	88	CD44^+^CD24^-/low^	No statistically significant associations were found (P-values > 0.05)	For CD44^+^CD24^-/low^, it was above 10%.	IHC	ab51037 for CD44 and ab31622 for CD24	Eighty-two patients were on adjuvant chemotherapy.
**Yan−Xi Liu, (** 27 **)**	140	CD44^+^CD24^-/low^	Compared to AR^+^ TNBC, it is associated with higher tumor grades.	For CD44^+^CD24^-/low^, it was above 10%.	IHC	156−3C11 for CD44, and Ab2−SN3b for CD24	One hundred twenty-three patients were on chemotherapy.
**Hui Wang, (** 28 **)**	145	CD44^+^CD24^-/low^	It is positively associated with AR expression.	For CD44^+^CD24^-/low^, it was above 10%.	IHC	ab51037 for CD44, and ab31622 for CD24	None of the patients were on targeted therapy, radiotherapy, chemotherapy, and adjuvant endocrine treatment.
**Weiyan Zou, (** 29 **)**	51	CD44^+^CD24^-/low^	It is associated with advanced tumor stage, large tumor size, and increased lymph node metastasis.	N/A	IHC	ab51037 for CD44 and ab31622 for CD24	N/A
**Nazia Riaz, (** 35 **)**	197	CD44^+^CD24^-/low^	CD44^+^CD24^-/low^ is correlated with the lack of AR expression.	N/A	IHC	M7082 for CD44, and MS1279 for CD24	All of the patients were on standard radiotherapy/chemotherapy.

IHC, immunohistochemistry; N/A, not available; and AR, androgen receptor.

Regarding the prognostic value of CD44 in TNBC patients, CD44 has been associated with inferior DFS in affected patients (**Table 3**). Regarding the prognostic value of the CD44^+^CD24^-/low^ phenotype in TNBC patients, this phenotype has been associated with inferior OS and DFS in affected patients (**Table 3** for a better elucidation, refer to the discussion). Regarding the cross-talk between CD44^+^CD24^-/low^ phenotype with TNBC development, this phenotype has been associated with epithelial cadherin (E-cadherin) loss, overexpressed CD146, upregulated vimentin, increased tumor necrosis, elevated Ki-67 level, higher EGFR expression, and downregulated claudin3/4/7 (**Table 4**).

**Table 3:** The prognostic value of CD44 and CD44^+^CD24^-/low^ phenotype in TNBC patients.

First author, year	Endpoint	Number of patients	Studied factor	Prognostic value	HR, CI 95%, and P-value	Follow-up time	Previous treatment
**Francesca Collina, (** 25 **)**	DFS	143	CD44	Its overexpression is associated with worse DFS.	Not provided	About 90 months	N/A
**Fang Yang, (** 26 **)**	PFS	88	CD44^+^CD24^-/low^	It is not statistically associated with PFS.	1.74, (0.73-4.13), and 0.211	72 months	Eighty-two patients were on adjuvant chemotherapy.
**Yan−Xi Liu, (** 27 **)**	RFS	123	CD44^+^CD24^-/low^	It is not statistically associated with RFS.	2.17, (0.76−2.74), and 0.006	68 monthes	With chemotherapy
**Yan−Xi Liu, (** 27 **)**	Breast cancer-specific survival	123	CD44^+^CD24^-/low^	It is not statistically associated with breast cancer-specific survival.	2.30, (0.95−2.84), and 0.003	68 months	With chemotherapy
**Yan−Xi Liu, (** 27 **)**	RFS	31	CD44^+^CD24^-/low^	It is not statistically associated with RFS.	1.68, (1.14−3.07), and 0.115	68 months	No previous chemotherapy
**Yan−Xi Liu, (** 27 **)**	Breast cancer-specific survival	31	CD44^+^CD24^-/low^	It is not statistically associated with breast cancer-specific survival.	1.72, (0.88−2.74), and 0.092	68 months	No previous chemotherapy
**Hui Wang, (** 28 **)**	DFS	145	CD44^+^CD24^-/low^	It is not statistically associated with DFS.	2.38, (0.90−6.33), and 0.081	76 months	None of the patients were on targeted therapy, radiotherapy, chemotherapy, and adjuvant endocrine treatment.
**Hui Wang, (** 28 **)**	OS	145	CD44^+^CD24^-/low^	It is associated with OS.	4.38, (1.57−12.18), and 0.005	76 months	None of the patients were on targeted therapy, radiotherapy, chemotherapy, and adjuvant endocrine treatment.
**Weiyan Zou, (** 29 **)**	OS	51	CD44^+^CD24^-/low^	Regardless of lymph node metastasis, it is associated with OS.	Not provided	About 70 months	N/A
**Weiyan Zou, (** 29 **)**	DFS	51	CD44^+^CD24^-/low^	Regardless of lymph node metastasis, it is associated with DFS.	Not provided	About 70 months	N/A

DFS, disease-free survival; PFS, progression-free survival; RFS, relapse-free survival; OS, overall survival; HR, hazard ratio; CI, confidence interval.

**Table 4:** The studied cross-talk with the CD44^+^CD24^-/low^ in TNBC cells.

First author, year	Studied factor	Studied cross-talk with the desired factor	Effect on TNBC cells
**Min Hye Jang, (** 33 **)**	CD44^+^CD24^-/low^	Compared to TNBC cells without CD44^+^CD24^-/low^, it is associated with E-cadherin loss, upregulated CD146, and overexpressed vimentin.	It can promote the EMT process.
**Yan−Xi Liu, (** 27 **)**	CD44^+^CD24^-/low^	Compared to AR^+^ TNBC, it is associated with increased Ki-67, E-cadherin loss, upregulated vimentin, and decreased claudin3/4/7.	It can promote metastasis.

E-cadherin, epithelial cadherin; EGFR, epidermal growth factor receptor; and EMT, epithelial-mesenchymal transition.

### The Risk of Bias in Included Studies

Based on the six items of Hayden et al. guidelines, the quality of the included studies was evaluated (**Table 1**). The study participation and attrition items were scored well according to the guideline. The main risk areas were prognostic factor measurement and analysis.

## Discussion

The following sections are aimed to critically review the results of the including studies about the prognostic value of CD44 and CD44^+^CD24^-/low^ phenotype, their association with the clinicopathological features of TNBC patients, and their associations with the EMT process, metastasis, chemoresistance, and tumor microenvironment of TNBC cells according to the preclinical studies to present a better picture of CD44 and CD44^+^CD24^-/low^ phenotype in TNBC cells. Finally, we briefly review the current-evaluated preclinical approaches in targeting CD44 to inhibit TNBC development.

### CD44

Collina et al. have reported that TNBC patients with upregulated expression of cytoplasmic CD44 might demonstrate worse PFS compared to the TNBC patients with low CD44 expression (25). The expression of CD44 has been substantially associated with higher tumor grade, lymph node metastasis, and advanced tumor stage in TNBC patients (29). Consistent with this, CD44 has been implicated in promoting lymphovascular invasion in TNBC patients (32). In line with this, there has been a remarkable association between CD44 expression and tumor metastasis in TNBC patients (25). Therefore, CD44 can be associated with advanced tumor stage, higher tumor grade, tumor metastasis, and lymphatic involvement in TNBC patients. Besides, CD44 overexpression might indicate an inferior prognosis in TNBC patients.

It has been reported that most TNBC cell lines are CD44-positive, making this factor a promising target for treating TNBC (36). A better understanding of its underlying cross-talk in chemoresistance, immunosuppression, and tumor migration is critical for treating TNBC patients. In TNBC cells, CD44 has been implicated in the upregulation of tumoral PD-L1 (15). Moreover, PD-L1 is required for the expression of CD44 in TNBC. Indeed, Lotfinejad et al. have indicated that PD-L1 silencing remarkably downregulates the expression of CD44 in TNBC cells (37). Zhang et al. have also reported a positive correlation between tumoral PD-L1 and CD44 in lung adenocarcinoma (38). It is well-established that PD-L1 can impede the development of anti-tumoral immune responses and result in tumor development (39). A recent meta-analysis has indicated a strong association between tumor-infiltrating lymphocytes and tumoral PD-L1 in TNBC patients (40).

In breast cancer patients, Zheng et al. have reported a strong positive association between CD44 and EGFR (41). Compared to AR^+^ TNBC cells, CD44^+^CD24^-/low^ TNBC cells can upregulate EGFR expression (27). With the upregulation of EGFR in some TNBC cells, targeting EGFR *via* cetuximab administration has been a promising strategy for treating TNBC patients. Wenyan et al. have shown that delivering CD44-siRNA into EGFR^+^ TNBC cells can enhance the sensitivity of EGFR^+^ TNBC cells to cetuximab (42). EGFR and mucin 1 (MUC1), which are present in 90% of TNBC cells, can establish multiple immunosuppressive positive loops, resulting in the recruitment of myeloid-derived suppressor cells (MDSCs), leading to an immunosuppressive tumor microenvironment (43). Of interest, MUC-1 can also upregulate PD-L1 and promote tumor growth (44). Thus, in this intertwined network, CD44 is a critical factor for inducing immunosuppressive tumor microenvironment and tumor growth.

Regarding drug resistance, CD44-siRNA transfection can decrease clonogenicity and downregulate the expression of VEGF, MMP-9, and CXCR4 in MDA-MB-468 cells. Furthermore, the combination therapy of CD44-siRNA and doxorubicin has substantially decreased the half-maximal inhibitory concentration (IC50) of doxorubicin (45). Cheng et al. have shown that the doxorubicin-resistant MDA−MB−468 cells can considerably express CD44, and inhibiting STAT-3 can decrease the CD44^+^ cell population and enhance the chemosensitivity of MDA−MB−468 cells *via* the STAT-3/Oct-4/c-Myc pathway (46). There is growing evidence about the adverse effect of CD44 on the chemosensitivity of tumoral cells. In MCF-7/Adr cells, the interaction of CD44 with hyaluronan can activate the downstream signaling pathway of Erb-B2 receptor tyrosine kinase 2 (ErbB2), the PI3K pathway, which leads to the upregulation of MDR-1. Of interest, the stimulation of the PI3K signaling pathway results in hyaluronan production, leading to the establishment of an auto-inductive chemoresistant loop in breast cancer cells (47). Bourguignon et al. have shown that the interaction of hyaluronan with CD44 can stimulate the Nanog, leading to the upregulation of MDR-1 in STAT-3 dependent fashion. Moreover, hyaluronan interaction with CD44 has been implicated in efflux chemotherapeutic agents by facilitating the interaction of ankyrin with MDR-1 in tumoral cells (17). CD44 has also been implicated in promoting Nanog, metastasis, and tumorgenicity in head and neck squamous cell carcinoma (48). Moreover, it has been reported that the CD44 activation can upregulate Nanog and subsequently repress apoptosis in tumor cells (49). Collectively, CD44 might promote immunosuppressive tumor microenvironment, tumor growth, tumor migration, and chemoresistance in TNBC cells.

### The CD44^+^CD24^-/Low^ Phenotype in TNBC Patients: Untangling the Controversial Results

#### The CD44+CD24-/Low Phenotype and Its Prognostic Value in TNBC Patients

Zou et al. have reported that TNBC patients with the phenotype of CD44^+^CD24^-/low^ have remarkably worse DFS and OS compared to the TNBC patients without the CD44^+^CD24^-/low^ phenotype (29). Besides, TNBC patients with CD44^+^CD24^-/low^ phenotype have experienced worse OS compared to CD44^−^/CD24^−^ patients (HR = 4.38, CI 95%: 1.57−12.18, P-value = 0.005). However, compared to CD44^−^/CD24^−^ TNBC patients, there has been no statistically significant association between DFS and CD44^+^CD24^-/low^ phenotype (P-value = 0.081) (28). Compared to luminal A breast cancer patients, treatment-naïve CD44^+^CD24^-/low^ TNBC patients have not have statistically significant worse relapse-free survival (RFS) and breast cancer-specific survival (both P-values > 0.05) (27). Although CD44^+^CD24^-/low^ TNBC patients have not had statistically poor PFS in comparison to the CD133^+^ and/or aldehyde dehydrogenase 1 family member A1^+^ (ALDH1A1^+^) ones, the CD44^+^CD24^-/low^ and/or ADLH1A1^+^ TNBC ones have had worse PFS in comparison with their counter partner TNBC patients (HR = 2.81, CI 95%: 1.26-6.24, P-value = 0.011) (26).

These seemingly conflicting results might be stemmed from the different references and relatively small sample sizes in these studies. In comparison with the CD44^−^/CD24^−^ TNBC patients, there have been no statistically significant results for determining DFS of CD44^+^CD24^-/low^ TNBC patients (P-value > 0.05) (28). Liu et al. have conducted the comparison between the luminal A patients with the CD44^+^CD24^-/low^ TNBC patients, which have not led to statistically significant results regarding the RFS and breast cancer-specific survival (both P-values > 0.05) (27). In comparison with CD133^+^ and/or ALDH1A1^+^ TNBC patients, there have been no statistically significant results for determining the PFS of CD44^+^CD24^-/low^ TNBC patients (P-value > 0.05) (26). Indeed, the comparison between the TNBC patients expressing CD44^+^CD24^-/low^ phenotype with the TNBC patients not expressing CD44^+^CD24^-/low^ can determine the prognostic value of CD44^+^CD24^-/low^ phenotype in TNBC patients. Given this, regardless of lymph node metastasis, the CD44^+^CD24^-/low^ phenotype can worsen DFS and OS of TNBC patients compared to TNBC patients without the CD44^+^CD24^-/low^ phenotype (29). In line with this, breast cancer patients with high level of CD44^+^CD24^-/low^ have demonstrated worse DFS and OS compared to breast cancer patients with low level of CD44^+^CD24^-/low^ (HR = 1.890, CI 95%:1.217-3.464, P-value = 0.015, and HR = 1.92, CI 95%: 1.248-3.586, P-value = 0.017, respectively) (50). Thus, the CD44^+^CD24^-/low^ phenotype can be associated with inferior survival in TNBC patients.

#### The CD44^+^CD24^-/Low^ Phenotype and Its Association With Clinicopathological Features of TNBC Patients

The CD44^+^CD24^-/low^ phenotype expression has been frequent in basal-like neoplasms than in non-basal-like neoplasms (33). Consistent with this, Riaz et al. have shed light on a correlation between CD44^+^CD24^-/low^ phenotype and basal-like TNBC in chemotherapy and radiotherapy-experienced basal-like TNBC patients (35). Among the CD44/CD24 phenotypes, CD44^+^CD24^-/low^ has been associated with more aggressive TNBC regarding the tumor size, TMN stage, and lymph node metastasis (29). Consistent with this, the CD44^-^/CD24^+^ phenotype has associated with less lymphovascular invasion in TNBC patients (32). Besides, the CD44^+^CD24^-/low^ phenotype has been more frequent in high-grade TNBC cells (33). With the sample size of 67 TNBC patients, Chang et al. have failed to establish any statistically significant associations between CD44^+^CD24^-/low^ phenotype with TNM stage, tumor grade, lymph node metastasis among the CD44/CD24 phenotypes (all P-values > 0.05) (34). These conflicting results might be due to the relatively small sample size of Chang’s study. Therefore, CD44^+^CD24^-/low^ phenotype can be associated with tumor size, TMN stage, lymph node metastasis, and tumor grade in TNBC patients.

#### The CD44^+^CD24^-/Low^ Phenotype in Treatment-Naïve and Treated Patients and Its Cross-Talk With Chemoresistance and Metastasis

Among the different CD44/CD24 phenotypes, CD44^+^CD24^-/low^ cells have expressed a substantial AR in TNBC patients without previous chemotherapy and radiotherapy (28). However, in treated TNBC patients with standard chemotherapy and radiotherapy, the CD44^+^CD24^-/low^ phenotype is inversely correlated with AR expression (35). Indeed, AR expression has been associated with improved OS and breast cancer-specific survival in treated TNBC patients (35). Consistent with this, the CD44^+^CD24^-/low^ TNBC cells have exhibited a more aggressive histological pattern, high Ki67 score, increased vimentin, and upregulated EGFR, decreased E-cadherin, and downregulated claudin-3/4/7 compared to AR^+^ TNBC cells (27). Given this, the CD44^+^CD24^-/low^ phenotype might decrease the AR expression and develop chemoresistance following chemo-and radiotherapy in TNBC patients (see below). Consistent with our observed results, Lehmann et al. have indicated that mesenchymal and mesenchymal stem-like subtypes, which are substantially enriched for the Wnt/β-catenin signaling pathway, predominantly stimulate the EMT and express CD44^+^CD24^-^ phenotype. Mesenchymal and mesenchymal stem-like subtypes have been associated with inferior 5-year distant metastasis-free survival. Besides, the mesenchymal subtype has been associated with the inferior RFS of affected patients, and this subtype overexpresses proliferation-related genes. However, TNBC patients with luminal androgen receptor subtypes have shown improved RFS compared to patients with other subtypes (3).

Jang et al. have reported remarkable associations between CD44^+^CD24^-/low^ phenotype with E-cadherin loss, CD146, and vimentin expression in TNBC cells (33). Recently, Vikram et al. have indicated that the CD44^+^CD24^-/low^ phenotype can lead to the overexpression of the EMT/metastatic markers, e.g., Nanog and sex-determining region Y-related HMG box 2 (SOX2), in MDA-MB-231 cells. Indeed, the CD44^+^CD24^-/low^ phenotype has been positively associated with tumor growth and migration in TNBC cells (51). Following doxorubicin treatment, doxorubicin-resistant MDA-MB-231 cells have substantially upregulated the CD44^+^CD24^-/low^ phenotype compared to wild-type cells (52). Besides the TNBC cells, growing evidence indicates that the CD44^+^CD24^-/low^ phenotype can promote EMT and chemoresistance in other cancers. In oral squamous cell carcinoma, the CD44^+^CD24^-/low^ phenotype has promoted colony formation, tumor migration, and the expression of drug transporters, which can facilitate the EMT process and chemoresistance (53).

### Lessons From the Past and the Road Ahead

Targeted therapy has become an ever-increasingly appealing approach for treating cancer patients. Based on our discussion, TNBC cells, in response to current chemotherapy, can lead to chemoresistance and tumor relapse, which the cancer stem cells have been implicated in promoting that. Therefore, it is pressingly needed to eradicate the cancer stem cells from tumor bulk. The following discussion intends to present novel paradigms for targeting CD44, as an essential cancer stem cell factor, in TNBC.

The miR-based therapy and small interfering RNA (siRNA)-based therapy can post-transcriptionally alter the expression of CD44. Preclinical studies have supported their efficacy in eradicating tumor cells. Vahidian et al. have demonstrated that the doxorubicin combination with CD44-siRNA can substantially decrease tumor growth, metastasis and increase apoptosis in MDA-MB-468 cells. Besides, CD44-siRNA has considerably decreased the IC50 of doxorubicin in MDA-MB-468 cells (45). In line with this, Van Phuc et al. have shown that the CD44^+^CD24^-^ tumoral cells are resistant to doxorubicin, and targeting CD44 can substantially increase the sensitivity of breast cancer cells to doxorubicin (54). Eameema et al. have developed a drug delivery vehicle, which binds to CD44 *via* its anti-CD44 human antibody and delivers paclitaxel and salinomycin. They have demonstrated that this nanoparticle-based vehicle can specifically target CD44^+^ MDA-MB-231 cells and effectively eradicate the tumoral cells (55). Fu et al. have shown that the delivery of CD44-siRNA can substantially enhance the cetuximab sensitivity of TNBC cells, and the combined delivery of CD44-siRNA with cetuximab treatment can remarkably decrease tumor volume in mice bearing TNBCs (42). Targeting CD44 in TNBC cells has also been associated with increased survival of mice-bearing tumors, decreased tumor burden, and suppressed bone metastasis in aminal models (56). Consistent with these, the combined downregulation of CD44 with doxorubicin administration has considerably decreased tumor volume compared to animal models treated with doxorubicin (57). A liposomal-based vehicle, which delivers miR-34a to breast cancer cells, can downregulate ZEB1, Bmi1, and CD44 expression and eradicated breast cancer cells (58). Ahir et al. have designed a mesoporous silica nanoparticle vehicle, covered hyaluronic acid, to deliver miR-34a and antisense-miR-10b into TNBC cells. Their *in vitro* and *in vivo* results have shown promising outcomes regarding inhibition of tumor growth and metastasis (59). Al-Othman et al. have demonstrated that the transfection of miR-328-3p, which has been upregulated following the treatment of TNBC with 5α-dihydrotestosterone, can reduce CD44 expression and tumor migration in TNBC. Based on their study, 5α-dihydrotestosterone can downregulate CD44 expression *via* binding the AR/5α-dihydrotestosterone to CD44 promoter or upregulating the expression of miR-328-3p, which can inhibit post-transcriptionally decrease the expression of CD44 (60).

Moreover, the recent advances in immunotherapy have provided ample opportunities to ameliorate the prognosis of TNBC patients. Immunotherapeutic approaches are focused on stimulating anti-tumoral immune responses to reject tumoral cells. The PD-L1/programmed cell death protein 1 (PD-1) axis is a well-known inhibitory immune checkpoint axis that can substantially attenuate anti-tumoral immune responses (39, 61). This axis can be established between tumoral cells and effector immune cells and shield the tumoral cells from anti-tumoral immune responses (40). Recently, Lotfinejad et al. have shown that inhibiting tumoral PD-L1 can substantially decrease CD44 expression in TNBC cells (37). Besides, inhibiting CD44 has been associated with decreased expression of PD-L1 in TNBC cells (15). Consistent with these, it has been shown that selective inhibition and activation of the Wnt signaling pathway, which is enriched for cancer stem cell markers, can remarkably downregulate and upregulate PD-L1 expression in TNBC cells (62, 63). Thus, this positive association between CD44 and PD-L1 might provide the rationale for investigating the effect of monoclonal PD-L1/PD-1 antibodies administration on the CD44 expression and stemness of TNBC cells in affected patients.

The current systematic review has several strengths. First, given the controversial results of clinical studies accumulated between 2014 to 2020 regarding the prognostic values of the CD44^+^/CD24^-^ phenotype in TNBC patients, our study has clarified its prognostic value in TNBC patients. Second, besides its prognostic value, we have clarified its clinicopathological significance in TNBC patients, which enables clinicians to determine the course of TNBC in affected patients. However, our systematic review has some limitations, as well. First, we only included the clinical studies that were published in English. Second, the population of our included studies was geographically and, presumably, ethnically diverse, which can lead to increase heterogeneity among the included studies. Third, the currently available evidence has used IHC staining for detecting protein expression; in light of the recent advances in mass-cytometry technologies, there might be a need to investigate the impact of CD44 and CD44^+^CD24^-^ at the single-cell levels.

## Conclusion

Since cancer stem cells are one of the daunting challenges of treating TNBC patients, identifying and categorizing them can provide valuable insights for targeted therapies. The current systematic review has demonstrated that CD44 and CD44^+^CD24^-/low^ phenotype are associated with inferior prognosis in TNBC patients, and they are correlated with advanced tumor stage, tumor size, higher tumor grade, tumor metastasis, and lymphatic involvement in TNBC patients. These cancer stem cell factors can lead to chemoresistance, EMT activation, induction of immunosuppressive tumor microenvironment, and tumor growth in TNBC cells. The combined downregulation of CD44 and the administration of chemotherapeutic agents, e.g., doxorubicin, has shown promising results in preclinical studies. Besides, the combination of CD44-siRNA and specific tumor-suppressive miRs has been associated with enhanced chemosensitivity of TNBC cells to chemotherapeutic agents and decreased tumor growth both *in vivo* and *in vitro* studies. Therefore, siRNA/miR-based gene therapy and their combination with chemotherapeutic agents can provide ample opportunities to improve the prognosis of TNBC patients.

## Data Availability Statement

The original contributions presented in the study are included in the article/supplementary material. Further inquiries can be directed to the corresponding authors.

## Author Contributions

MA and NeH contributed to the study selection. MA developed the systematic search, interpreted the results, and wrote the majority of the manuscript. AD and NiH have contributed to the assessment of included studies. NKA, ZA, PL, and OB have extracted the data from the included studies. NS and BB have supervised the project. All authors contributed to the article and approved the submitted version.

## Funding

This study is supported by the Research Center for Evidence-Based Medicine, Tabriz University of Medical Sciences, Tabriz, Iran (number: 67306).

## Conflict of Interest

The authors declare that the research was conducted in the absence of any commercial or financial relationships that could be construed as a potential conflict of interest.

## Publisher’s Note

All claims expressed in this article are solely those of the authors and do not necessarily represent those of their affiliated organizations, or those of the publisher, the editors and the reviewers. Any product that may be evaluated in this article, or claim that may be made by its manufacturer, is not guaranteed or endorsed by the publisher.
